# Individual Differences in Discriminatory Fear Learning under Conditions of Ambiguity: A Vulnerability Factor for Anxiety Disorders?

**DOI:** 10.3389/fpsyg.2013.00298

**Published:** 2013-05-28

**Authors:** Inna Arnaudova, Angelos-Miltiadis Krypotos, Marieke Effting, Yannick Boddez, Merel Kindt, Tom Beckers

**Affiliations:** ^1^Department of Clinical Psychology and Cognitive Science Center Amsterdam, University of Amsterdam, Amsterdam, Netherlands; ^2^Department of Psychology, KU Leuven, Leuven, Belgium

**Keywords:** individual differences, selective fear-conditioning, discriminatory fear learning, anxiety, cue competition

## Abstract

Complex fear learning procedures might be better suited than the common differential fear-conditioning paradigm for detecting individual differences related to vulnerability for anxiety disorders. Two such procedures are the *blocking* procedure and the *protection-from-overshadowing* procedure. Their comparison allows for the examination of discriminatory fear learning under conditions of ambiguity. The present study examined the role of individual differences in such discriminatory fear learning. We hypothesized that heightened trait anxiety would be related to a deficit in discriminatory fear learning. Participants gave US-expectancy ratings as an index for the threat value of individual CSs following blocking and protection-from-overshadowing training. The difference in threat value at test between the protected-from-overshadowing conditioned stimulus (CS) and the blocked CS was negatively correlated with scores on a self-report tension-stress scale that approximates facets of generalized anxiety disorder (GAD), the Depression Anxiety Stress Scale-Stress (DASS-S), but not with other individual difference variables. In addition, a behavioral test showed that only participants scoring high on the DASS-S avoided the protected-from-overshadowing CS. This observed deficit in discriminatory fear learning for participants with high levels of tension-stress might be an underlying mechanism for fear overgeneralization in diffuse anxiety disorders such as GAD.

## Introduction

According to a diathesis-stress model of anxiety disorders, only individuals with certain ingrained vulnerabilities will develop an anxiety disorder following a frightening or traumatic conditioning experience (Mineka and Zinbarg, 2006). The underlying idea of this model is that particular personality traits may predispose some individuals to enhanced fear conditionability (ease of associative fear learning; Otto et al., 2007). That is, following a real-life conditioning event, vulnerable individuals are suggested to have a maladaptive fear response, which serves as the foundation for the development of an actual anxiety disorder. Thus, an important step to truly grasping the etiology of anxiety disorders is identifying individual difference variables that influence fear conditionability in a laboratory setting (i.e., Eysenck, 1976; Zinbarg and Mohlman, 1998; Lissek et al., 2005; Mineka and Zinbarg, 2006). Despite considerable efforts to do so, research has yielded mixed empirical results (Joos et al., 2012).

Imperfections of current research methods have been pinpointed as part of the reason behind the inconclusiveness of the findings (Lissek et al., 2005). For example, one crucial aspect of conditioned fear responding that might be particularly prone to effects of individual difference variables, behavioral avoidance, has often been overlooked in research so far (Beckers et al., 2013). In addition, the commonly used *differential fear-conditioning* paradigm has been criticized as a model for pathological fear learning (Lissek et al., 2006; Mineka and Oehlberg, 2008; Beckers et al., 2013). In this paradigm, a neutral stimulus (*conditioned stimulus*, CS+) is repeatedly paired with an aversive outcome (*unconditioned stimulus*, US; e.g., shock), resulting in a conditioned fear-like reaction to the CS. This is revealed by increased US-expectancy ratings and physiological reactivity upon presentation of the CS+. A second neutral stimulus (CS−) is never followed by the US, thus acting as a safe signal in the paradigm. A comparison of fear responding to the CS+ and the CS− allows for the assessment of *discriminatory fear learning*. Reduced discriminatory fear learning is considered maladaptive, because in such case responding is not based upon actual stimulus contingencies (Lissek et al., 2005).

This procedure essentially represents a hedonically strong situation: the CS+ clearly signals danger, while the CS− clearly signals safety (Lissek et al., 2006). Because of this threat unambiguity, responses can be expected to be relatively uniform across individuals (Lissek et al., 2006). The lack of ambiguity in this procedure obstructs the examination of interindividual variability in fear learning: mostly everyone will exhibit fear upon confrontation with the CS+ and inhibit fear upon confrontation with the CS− (Lissek et al., 2006; Beckers et al., 2013). A number of studies have actually failed to find an effect of trait anxiety (a known vulnerability factor for anxiety disorders; Spielberger and Gorsuch, 1983) on differential fear conditioning (e.g., Joos et al., 2012; Torrents-Rodas et al., 2013; but see Baas et al., 2008; Indovina et al., 2011; Gazendam et al., 2013). When comparing clinical with non-clinical populations, reduced discriminatory fear learning has been sometimes successfully observed among participants with anxiety disorders (for a review, see Lissek et al., 2005). From these studies, however, it is not clear if discriminatory fear learning is involved in the etiology or the maintenance of the disorders, because patients are tested after they have been diagnosed with an anxiety disorder (Beckers et al., 2013).

The use of a weaker or a more ambiguous assessment situation might be better suited to study individual differences in fear conditioning, because it increases the variance of individual responses and will make the proposed maladaptive responses of vulnerable individuals more apparent (Lissek et al., 2006; Beckers et al., 2013). For example, it has been observed that relative to low-neuroticism participants, participants with high neuroticism showed increased avoidance to generalization stimuli derived from a CS+ (Lommen et al., 2010). Generalization stimuli do not have a direct link to the US; their threat value is estimated from their perceptual similarity to the CS+, which makes them essentially ambiguous. Chan and Lovibond (1996) used another ambiguous assessment method, a *conditioned inhibition paradigm* (A+ training intermixed with AB− training), and found that individuals who were high in trait anxiety and were also unaware of stimulus contingencies in the task showed an expectancy bias (increased US-expectancy) for all CSs. These results provide empirical evidence for the conceptual argument of Lissek et al. (2006) that individual differences are particularly likely to be observed in weak or ambiguous testing situations.

Following this reasoning, the optimal assessment of individual differences in discriminatory fear learning requires a comparison of an ambiguous danger and an ambiguous safe signal. This can be achieved through the use of a *selective fear-conditioning paradigm*, where multiple stimuli compete for behavioral control of the fear response, thus creating some level of ambiguity. For example, a selective conditioning procedure called *protection from overshadowing* can be regarded as the ambiguous counterpart for the learning of a danger signal (CS+) in differential fear conditioning. In protection from overshadowing, one CS (C) is presented without being followed by the US in a first *elemental conditioning* phase (C−). In a second *compound conditioning* phase, C is presented together with another CS (D) to make up a compound of two CSs (CD), which is followed by the US (CD+). Following a protection-from-overshadowing procedure (C− then CD+) in associative learning tasks, heightened responding is generally assigned to the *protected-from-overshadowing* stimulus D relative to a situation where only CD+ training is given (Vandorpe and De Houwer, 2005). The fact that C is not followed by the US in selective conditioning, when presented alone, suggests that D is probably dangerous (with a higher threat value), given that the chances of the US are clearly increased by adding D to C. However, the high threat status of D remains somewhat ambiguous and can only be inferred, because D is never observed in isolation before test.

In order to analogously create an ambiguous signal for relative safety, one CS (A) can be repeatedly followed by a US in a first phase of conditioning (A+). In a subsequent compound conditioning phase, A can be presented together with another CS (B) to make up a compound of two CSs (AB), which is also followed by the US (AB+). Following such *blocking* procedure (A+ then AB+) in associative learning tasks, it is typically found that responding to the *blocked* CS B is reduced relative to a situation where only AB+ training is presented (Kamin, 1969; Dickinson et al., 1984). The blocking effect has been observed in a variety of learning procedures in diverse species (see Haselgrove and Evans, 2010, for an overview). Thus, in a conditioning procedure, the fact that A is followed by the US when presented alone suggests that B is probably safer (has a lower threat value) than a protected-from-overshadowing D, given that the chances or the intensity of the US following the AB compound are not increased by B. Still, the relative safety of B in comparison to D remains ambiguous and can only be inferred, given that B is never observed in isolation before test (both B and D are only ever presented in a compound that is always followed by the US; Beckers et al., 2013). Individual differences in such selective learning of relative safety might therefore be readily observed. In line with this idea, it has indeed been shown that trait anxiety is correlated with reduced blocking (thus, impaired safety learning for a blocked stimulus; Boddez et al., 2012). Therefore, a selective discrimination learning procedure, where protection-from-overshadowing and blocking training are combined, allows examining discriminatory fear learning under conditions of ambiguity and uncovering individual differences therein.

Since the early years of fear-conditioning research, most attention has been paid to the role of trait anxiety in conditionability (e.g., Spence, 1964), specifically in relation to deficient safety learning. Trait anxiety is usually assessed by means of the State and Trait Anxiety Inventory (STAI; Spielberger and Gorsuch, 1983), which has recently been questioned as a pure measure of dispositional anxiety and is now seen rather as a measure of general negative affect (Bieling et al., 1998; Grös et al., 2007; Bados et al., 2010). To address the lack of specificity of the STAI and other questionnaires, the Depression Anxiety Stress Scales (DASS; Lovibond and Lovibond, 1995) were developed. They measure three negative emotional states with good discriminative validity (Clara et al., 2001; Crawford and Henry, 2003): depression (loss of self-esteem and motivation; DASS-D), anxiety (physical arousal; DASS-A), and tension-stress (persistent tension and a low threshold for distress; DASS-S). The DASS-A has predictive validity for panic, phobia, and other anxiety disorders (Brown et al., 1997) and might be related to reactivity to threat. The DASS-S has been mainly linked to generalized anxiety disorder (GAD; Brown et al., 1997), thus possibly having a specific relationship with discriminatory fear learning [GAD patients experience chronic anxiety over a number of situations; American Psychiatric Association (APA), 2000]. DASS-S has recently been linked to worry (Szabó, 2011). Interestingly, worry has recently also emerged as a predictor for heightened conditionability (Otto et al., 2007; Gazendam and Kindt, 2012; Joos et al., 2012), making it crucial to discriminate the role of anxiety and tension-stress during fear conditioning. Other personality traits related to trait anxiety such as neuroticism and extraversion have also been implicated as potential sources for individual variability in fear learning (Eysenck, 1976) and this proposal has received partial support from a few studies (e.g., Frederikson and Georgiades, 1992; Pineles et al., 2009).

Disentangling the web of mixed results regarding these closely related personality characteristics and their influence on discriminatory fear learning under ambiguous conditions should allow a better understanding of vulnerability factors for anxiety disorders. In the present study, participants underwent blocking and protection-from-overshadowing training (see Table 1) and gave trial-by-trial US-expectancy ratings as indication of the threat value of each elemental and compound CS. The difference between the US-expectancy rating for the protected-from-overshadowing CS D and the blocked CS B (D minus B) at test was used as a measure of discriminatory fear learning (analogous to the difference score between CS+ and CS− typically used as index of learning in standard differential fear-conditioning studies, e.g., Joos et al., 2012). Based on the findings of Boddez et al. (2012), we hypothesized that trait anxiety should be associated with reduced discriminatory fear learning, mainly due to insufficient safety learning of the blocked CS. Other individual difference variables that have been implicated in conditionability were assessed as well for their unique contribution to disturbed discriminatory fear learning. Further, we examined the generalization of these effects to a behavioral task and across contexts. The behavioral task, in which participants chose between chocolate bars carrying symbolic representations of the blocked CS B and the protected-from-overshadowing CS D, was used to test whether individual differences can be observed in overt behavior as well. The role of test context (same or different as training context) was explored because of the lack of empirical data on the context specificity of learning following a selective fear-conditioning paradigm; we assumed that generalization across contexts might constitute another possible source of interindividual differences.

**Table 1:** **Conditioning contingencies**.

Type of training	Elemental	Compound	Context	Test
Blocking	A+	AB+	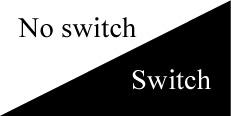	B−, D+, F−, A+, C−, E−
Protection from overshadowing	C−	CD+		D+, B−, F−, C−, A+, E−
Control	E−	EF−		

Letters represent CSs; − represents no US was administered; + represents US was administered.

## Materials and Methods

### Participants

A total of 68 participants from University of Amsterdam and the surrounding areas participated for course credits or a small monetary compensation (€ 7). Fourteen participants were excluded for lack of acquisition learning^1^. The remaining sample (20 males) had a mean age of 22.00 (SD = 4.48) years (see Table 2 for further demographics). All participants gave informed consent for their participation and the experimental procedure was approved by the Faculty Ethical Committee at the University of Amsterdam.

**Table 2:** **Mean and standard deviations (SD) for questionnaires, post-acquisition CS valence and US expectancy at CS test**.

Questionnaire	STAI-S	STAI-T	DASS-D	DASS-A	DASS-S	EPQ-N	EPQ-E	IUS
Mean	32.91	36.46	4.74	3.61	8.33	8.13	14.26	61.17
SD	7.34	7.19	6.08	3.86	6.78	5.37	3.67	17.30

**Post-acquisition CS valence**	**B**	**D**	**F**	**A**	**C**	**E**		

Mean	0.51	−1.56	1.85	−2.34	2.20	2.71		
SD	3.22	2.77	2.72	2.93	2.54	2.19		

**US expectancy at CS test**	**B**	**D**	**F**	**A**	**C**	**E**		

Mean	−0.29	3.07	−3.62	4.29	−3.53	−4.44		
SD	3.76	2.57	1.74	1.65	2.66	1.27		

### Stimuli and materials

Images of six colored three-dimensional geometrical objects as seen from four viewing angles (computer-generated) served as CSs: a yellow stick, a blue disk, a purple cylinder, a red plane, an orange cone, and a green cube. The longest dimension (height, diameter, or internal diagonal) of all objects was 60 mm. Objects appeared on the computer screen surrounded by a white frame, measuring 106 mm × 106 mm. They were centered on the screen with either an orange or blue background, counterbalanced across participants.

Conditioned stimulus assignment was partially counterbalanced across participants. The yellow stick, blue disk, and purple cylinder were counterbalanced to serve as elemental acquisition CSs A, C, or E. During the compound conditioning phase, the compound CSs were composed of the yellow stick and the red plane; the blue disk and the orange cone; the purple cylinder and the green cube (*de facto* counterbalanced to AB, CD, and EF, as a result of the counterbalancing of A, C, and E). In this phase, the two images, randomly assigned to the left or right part of the screen, appeared separated by 48 mm.

The US was an aversive 1-s 95-dB scream delivered through headphones.

### Assessments

#### US expectancy

Participants rated US expectancies by clicking with a mouse on a computerized 11-point Likert scale ranging from −5 (*certainly no scream*) to 5 (*certainly scream*). The validity of this measure to assess fear learning is reviewed extensively by Boddez et al. (2013).

#### Evaluative ratings

Valence ratings of CSs and the US were assessed on an 11-point Likert scale, with −5 indicating *very unpleasant* and 5 indicating *very pleasant*. The US was also rated on 5-category scales for intensity (*light*, *moderate*, *intense*, *enormous*, *unbearable*) and startlingness (*not*, *light*, *moderate*, *strong*, *very strong*).

#### Questionnaires

State and Trait Anxiety Inventory (Spielberger and Gorsuch, 1983; Dutch version by van der Ploeg, 2000) measures trait and state anxiety with 20 items each, with sum scores representing severity. The psychometric characteristics of the STAI are as follows: test-retest reliability 0.73–0.86 for STAI-T and 0.33 for STAI-S, internal consistency of 0.90 for STAI-T and 0.86–0.93 for STAI-S (Spielberger and Gorsuch, 1983) and excellent convergent validity across ethnic groups (Novy et al., 1993).

The 42-item DASS (Lovibond and Lovibond, 1995; Dutch translation by de Beurs et al., 2001) have good psychometric properties. Cronbach’s alphas for internal consistency of the three subscales DASS-D, DASS-A, and DASS-S are 0.97, 0.95, and 0.92, respectively (Antony et al., 1998).

Two scales of the Dutch Eysenck Personality Questionnaire (EPQ) measure neuroticism (22-item EPQ-N, Cronbach’s alpha = 0.87) and extraversion (19 item EPQ-E; Cronbach’s alpha = 0.85; Sanderman et al., 2012).

Responses to situations of ambiguity might also be influenced by dispositional intolerance of uncertainty. The 27-item Dutch version of the Intolerance of Uncertainty Scale shows good reliability with Cronbach’s alpha of 0.88 in a student sample (IUS; Freeston et al., 1994; Dutch translation by de Bruin et al., 2006).

#### Forced-choice behavioral test

Participants chose among 10 chocolate bars placed randomly in an open box by the exit of the experimental room. Five of the bars had a wrapping depicting the blocked CS B, while the rest had a wrapping representing the protected-from-overshadowing CS D; thus, participants’ choice reveals their preference for one or the other CS. This procedure was modeled after Blechert et al. (2007).

### Procedure

After signing an informed consent form, participants sat in front of a computer in a dimly lit room, where they were separated from the experimenter by a barrier. They filled in a computerized version of STAI-T and STAI-S.

On-screen instructions informed participants that their task was to predict the occurrence of a scream based on the objects presented on the screen. The US-expectancy rating scale and the usage of the mouse were explained. The experimenter repeated the on-screen instructions and asked participants to put on the headphones.

The selective conditioning procedure consisted of three phases: an elemental and a compound training phase, followed by a test phase (Table 1). During elemental training, three individual CSs were presented four times each, with one CS always being followed by the US (4 A+, 4 C−, and 4 E−). During compound training, participants viewed four presentations of three compound CSs, with two compound CSs being followed by the US (4 AB+, 4 CD+, and 4 EF−). Thus, across phases participants received blocking (A+ then AB+), protection-from-overshadowing (C− then CD+), and filler training (E− then EF−). The filler stimuli were used in order to indicate to participants that compound stimuli can occur without the US and to discourage participants from concluding that mere compoundness predicts US occurrence. Both learning phases occurred on the same orange or blue computer background (Context A).

In the test phase, six individual CSs were presented in a fixed, counterbalanced order that included the critical CSs B and D first, followed by all other elemental CSs (either B−, D+, F−, A+, C−, E−, or D+, B−, F−, C−, A+, E−). D and A trials were reinforced at test to prevent random ratings (Lovibond, 2003). Order was partially counterbalanced across participants in order to check for the influence of the reinforced test trials on the other ratings. Test trials occurred either on the same background (Context A) or on a background different from the acquisition context (Context B). Participants were randomly assigned to the context-switch or the no-context-switch condition.

Each elemental or compound CS presentation lasted 8 s. An active US-expectancy rating scale was available at the bottom of the screen during the first 5 s. If participants failed to confirm their rating by clicking the mouse button in this time frame, the pointer position at the end of the 5-s time frame of the current trial was recorded as an indication of their response^2^. Presentations of elemental or compound CS were randomized within the acquisition phases, with the restriction that no more than two identical trials were presented in succession. Inter-trial intervals (ITI) had an average duration of 20 s (15s, 20s, 25s). During ITIs and the last 3 s of CS presentation an inactive US-expectancy scale was present on the screen.

Following the test phase, participants took off the headphones and indicated for each elemental or compound CS presented during training whether it had been followed by the scream and the certainty in their response. After giving evaluative ratings for the CSs and the US, participants filled in the EPQ, the DASS, and the IUS. Then, participants performed the forced-choice behavioral test. Reinforcement of D at test might have potentially affected the choices made during the following behavioral test, but this should have occurred across participants, if anything acting to reduce the influence of individual differences on behavior.

### Data analysis

As counterbalancing factors (initial background, CS assignment, and test order) had no significant effects in preliminary analyses, the data were collapsed across them. Conditioning effects during elemental and compound training phases were analyzed using a 3 (trial type: A, C, E, or AB, CD, EF) by 4 (trial number: 1–4) repeated measures analyses of variance (ANOVAs). Repeated measures ANOVA was also used to examine the ratings of the six individual CSs at test, with a Bonferroni correction for pairwise comparisons. Greenhouse-Geisser corrections were applied when the assumption of sphericity was violated. In order to test for generalization of learning across contexts, context switch was entered as a between-subject variable in the repeated measures ANOVA.

To test for individual differences in discriminatory fear learning, we calculated correlations between scores on personality measures and the D-B difference score. The normal distribution of each variable was first examined with a Kolmogorov–Smirnov test. When the data were not normally distributed, Spearman’s correlations were used. Otherwise, Pearson’s *r* is reported. Participants scoring more than two standard deviations away from the mean on a personality measure were excluded for the analyses with that particular measure (*n* = 1 for STAI-S; *n* = 4 for DASS-D; *n* = 2 for DASS-A; *n* = 4 for EPQ-E; *n* = 1 for IUS). In order to check for generalization to a behavioral task, choice data were subjected to a chi-square test to evaluate deviation from random choice.

## Results

### Valence ratings

Mean ratings for the US were −2.80 (SD = 1.83) for valence, 2.76 (SD = 0.70) for intensity, and 2.89 (SD = 1.04) for startlingness, indicating that participants perceived the scream as aversive. US valence ratings were marginally correlated only with scores on STAI-T, *r* (54) = 0.27, *p*  = 0.047. Post-acquisition CS valence ratings can be seen in Table 2. As expected, CSs with higher threat values were given lower valence ratings compared to CSs with lower threat value.

### Conditioning effects

**Figure 1:**
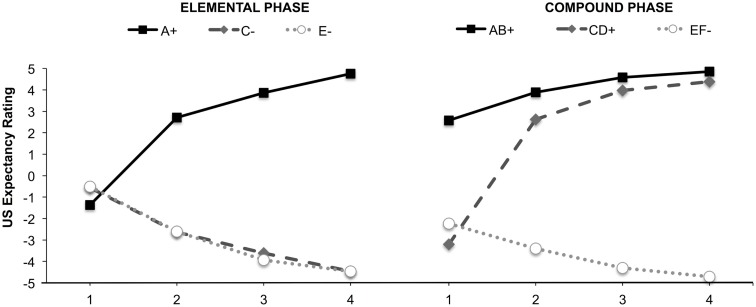
**US-expectancy rating during elemental (left panel) and compound conditioning (right panel)**.

Trial-by-trial US-expectancy ratings for the CSs during both learning phases can be seen in Figure 1. The ANOVAs revealed significant Trial type × Trial number interactions for both the elemental, *F*(3.89, 206.36) = 133.16, *p* < 0.001, $\eta_{p}^{2}=0.72$, and the compound phase, *F*(4.51, 238.89) = 81.50, *p* < 0.001, $\eta_{p}^{2}=0.61$. These results show that participants learned the contingencies between the specific CSs and the US across trials in both conditioning phases.

Unconditioned stimulus-expectancy ratings for the individual CSs at test can be found in Table 2. The six CSs elicited different ratings, *F*(2.99, 158.45) = 130.45, *p* < 0.001, $\eta_{p}^{2}=0.71$. All pairwise comparisons (each elemental CS with every other elemental CS) were significant (*p* < 0.01), except that US-expectancies for C were not significantly different from these for E and F (*p* > 0.10). The blocked stimulus B was rated significantly higher than the safe stimuli C, E, and F, which suggests that it remained ambiguous at test. The protected-from-overshadowing stimulus D was rated significantly lower than the dangerous stimulus A at test, which suggests it also remained somewhat ambiguous at test. However, the contrast between B and D was highly significant (*p* < 0.001). These results indicate that on average participants assigned higher threat value to the protected-from-overshadowing (relatively dangerous) CS D than the blocked (relatively safer) CS B, in line with expectations.

The main effect of CS on US-expectancy ratings was not modulated by context, *F* < 1. The test context did not affect ratings for B and D (*p* = 0.83). Our context manipulation did not affect the generalization of the assigned threat values.

### Individual differences in discriminatory fear learning

Contrary to our hypothesis, scores on the STAI-T did not correlate with overall discriminatory fear learning (D-B), ρ(54) = −0.15, *p* = 0.29. However, DASS-S scores did correlate with D-B, ρ(54) = −0.29, *p* = 0.03, and remained significant when controlling for DASS-A scores, ρ(49) = −0.29, *p* = 0.04. This suggests that high levels of persistent tension are linked to a deficit in discriminatory fear learning under ambiguity.

Remarkably, neither STAI-T, nor DASS-S, nor any of the other scores on personality measures were correlated to the difference between the US-expectancy rating between the two elemental CSs A and C (A minus C). The results confirm that interindividual differences in discriminatory fear learning are more readily detected for the ambiguous danger and safe signals than for non-ambiguous ones.

When looking at ratings for the individual CSs, STAI-T did not correlate with any of the US-expectancy ratings at test, although a trend was observed for the filler CS E, ρ(54) = 0.19, *p* = 0.07. The DASS-A emerged as the only marginally significant predictor of ratings for the ambiguous danger CS D, ρ(49) = −0.27, *p* = 0.05. A trend was observed for a correlation between the DASS-S and both CS B, *r* (54) = 0.26, *p* = 0.06, and CS D, ρ(54) = −0.26, *p* = 0.06. When controlling for DASS-A, the correlation between DASS-S and B became highly significant, *r* (49) = 0.45, *p* = 0.001, while its correlation with D became insignificant, ρ(49) = −0.03, *p* = 0.85. When controlling for DASS-S, the correlation between DASS-A and D also became insignificant, ρ(49) = −0.17, *p* = 0.22. The correlations between DASS-S and the other cues presented at test did not reach significance (all *p* > 0.10). Further, the correlation between DASS-S and the difference score between stimulus B and F at test, which might reflect more specifically the safety value of B, did not reach significance, *r* (54) = 0.159, *p* = 0.249.

No significant correlations or trends emerged between other personality measures (DASS-D, EPQ, and IUS) and the threat value assigned to any of the CSs, including the two stimuli of interest: the blocked stimulus B and the protected-from-overshadowing stimulus D. This suggests that the tension-stress scale of the DASS is best suited to capture individual differences in discriminatory fear learning under conditions of ambiguity; those differences moreover appear to occur predominantly in the selective learning of safety rather than danger.

### Forced-choice behavioral test

**Figure 2:**
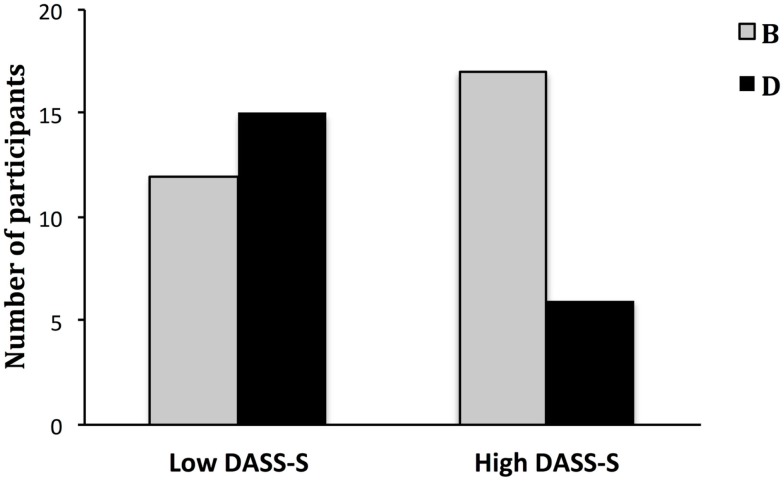
**Number of participants choosing a chocolate bar depicting either CS B or CS D in the forced-choice behavioral test according to DASS-S group**.

Generalization of the learned threat to overt behavior was examined through the total number of participants who showed a preference toward B. Participants did not show an overall preference for B over D during the forced-choice behavioral test, χ^2^(1) = 1.28, *p* = 0.26. Since only DASS-S emerged as a predictor of the extent of discrimination learning, a median split was performed to further analyze the data. The test showed that the two groups differed in their choice behavior, χ^2^(1) = 4.43, *p* = 0.04. The high DASS-S group chose B more often than D, χ^2^(1) = 5.26, *p* = 0.02 (Figure 2), whereas the low DASS-S group was indifferent, χ^2^(1) = 0.33, *p* = 0.56. This suggests that participants with high DASS-S scores actively avoided D.

## Discussion

This study examined individual differences in discriminatory fear learning under conditions of ambiguity. A reduction of discriminatory fear learning between a blocked CS and a protected-from-overshadowing CS was contrary to our hypothesis not related to any of the trait anxiety scores (STAI-T and DASS-A), but uniquely related to higher levels of tension-stress as measured by DASS-S. This result was driven mainly by increased threat value assigned to the blocked CS B, which suggests that these participants overestimate threat for ambiguous signals with relatively low threat value (i.e., overgeneralize threat from the AB+ compound trials to B). A tendency to overgeneralize was revealed for the high tension-stress group also in their performance during a behavioral task, where the high DASS-S participants showed more behavioral avoidance to a mere depiction of the protected-from-overshadowing CS D on a food item wrapping. This suggests that these participants judge ambiguous situations with the slightest hint of threat more readily as dangerous (i.e., a better-safe-than-sorry strategy). Such overgeneralization bias has been suggested as one of the underlying mechanisms of anxiety disorders with a generalized nature (e.g., Lissek and Grillon, 2010; Lissek et al., 2010).

This bias appears also to affect avoidance behavior under circumstances where there is no source of threat (as in the behavioral task). The observed behavioral pattern of the high tension-stress individuals can be seen as a sign of threat generalization toward an innocuous stimulus (a wrapping depicting a threatening CS).

The present study did not replicate the earlier observation by Boddez et al. (2012) of a significant correlation between trait anxiety as measured by STAI-T and threat value assigned to a blocked CS. The procedural differences between the two studies might partially explain the divergence. However, the nature of the STAI-T scale should be taken into account. Recent attempts to discriminate between depression and anxiety have prompted researchers to question the ability of STAI-T to specifically capture the concept of dispositional anxiety. Its items seem to reflect depression and general negative affect, rather than anxiety itself (Bieling et al., 1998; Grös et al., 2007; Bados et al., 2010). In contrast, the anxiety and stress scales of the DASS have been shown to capture factors of anxiety that are distinct from depressive symptoms (which are captured by the depression scale), with the DASS-A indexing in particular diagnostic approximations for phobias and panic disorder and the DASS-S capturing aspects of anxious distress that relate to more free-floating anxiety disorders such as GAD (Brown et al., 1997; Lovibond, 1998). Thus, the DASS scales offer the possibility to truly examine the divergent influence of three negative affective states upon discriminatory fear learning and to more readily draw conclusions about the link between vulnerability factors, discriminatory fear learning, and anxiety. Future research concerning individual differences in fear learning should utilize this aspect of the DASS scales to its advantage.

Only scores on the DASS-S scale were found to be linked to reduced discriminatory fear learning. One can argue that this relationship might be explained by an increased sensitivity of participants that score high on DASS-S to the aversive stimulus, but this is unlikely given the lack of correlation between US valence ratings and DASS-S scores, ρ(54) = −0.05, *p* = 0.72. Another possible interpretation of the results could be that participants with high tension-stress scores were less able to generalize from the last A+ trial in the elemental phase to the first AB+ trial in the compound phase and thus have learned more about the added stimulus B. Additional analyses, however, revealed no correlation between DASS-S scores on the one hand and expectancy ratings on the first AB+ trial, nor between DASS-S scores and generalization decrement (defined as the difference between responding on the final A+ trial and responding on the first AB+ trial), both *p*s > 0.7.

Depression Anxiety Stress Scale-Stress items correspond closely to the diagnostic criteria of GAD from the DSM-IV [American Psychiatric Association (APA), 2000] and the total score on the scale has recently been empirically linked to worry behavior, a core symptom of GAD (Lovibond, 1998; Szabó, 2011). The fact that worry has been shown to be related to increased conditionability (e.g., Otto et al., 2007) combined with the present results suggest that general tension-stress might be a vulnerability factor for GAD and maybe other diffuse anxiety disorders through its effect on discriminatory fear learning under conditions of ambiguity. More research with clinical and non-clinical samples is needed to confirm this possibility. The tentative results of this study suggest that in treatment, increasing the ability of GAD patients to discriminate between safer and more dangerous signals might be worthwhile in order to decrease behavioral avoidance and to improve functioning. Indeed, therapists increasingly come to recognize that learning about safety periods is a promising route in the treatment of GAD (e.g., Woody and Rachman, 1994; Fonteyne et al., 2009).

A secondary aim of this study was to examine the context specificity of selective learning. Our results show that selective learning generalizes across contexts. However, our context manipulation might have not been salient enough, as it consisted of only a screen background switch in the absence of any explicit instructions. Other limitations of this study include the studied sample (young university students), which puts generalization to the general population under question, and the use of correlational analyses and self-report data, which is known to be prone to demand characteristics.

Important questions remain for future research. The negative relation between selective discrimination learning and DASS-S scores might either be specific for threat-related situations (e.g., fear conditioning) or reflect a more general deficit in selective learning in people that are high in tension-stress. Future research might try to discriminate between a fear-specific versus a more general locus of the effect (e.g., by testing selective learning in neutral contingency learning tasks in relation to DASS-S scores). Also, learning theory and research suggest that several processes are involved in blocking and other forms of selective learning (Pearce and Bouton, 2001; De Houwer and Beckers, 2002; Shanks, 2010). An important challenge for future research is therefore to precisely determine the mechanisms that cause *variation* in selective (fear) learning. A deficit in selective attention (Le Pelley, 2004; Haselgrove et al., 2010) is one candidate process that could underlie the observed decrease in discrimination between protection from overshadowing and blocking in participants high in DASS-S (again, such lack of selective attention might be threat-specific or domain-general). Future research could examine this possibility by using attention measuring techniques (e.g., eye-tracking; Beesley and Le Pelley, 2011).

The present study offers empirical justification for the use of the selective fear-conditioning paradigm in the search for individual differences in discriminatory fear learning. A relationship between interindividual differences and discriminatory fear learning was observed only for ambiguous danger versus safety signals (D versus B) and not for unambiguous ones (A versus C). The present paradigm might therefore be useful for the examination of vulnerabilities to GAD. Future work should also strive toward establishing the unique contributions of anxiety, tension-stress, worry, and general negative affect to decreased discriminatory fear learning. Special attention needs to be paid to the tension-stress factor as this might predispose for the maladaptive expansion of threat toward innocuous stimuli.

## Conflict of Interest Statement

The authors declare that the research was conducted in the absence of any commercial or financial relationships that could be construed as a potential conflict of interest.

## Footnotes

^1^Excluded participants gave a positive US-expectancy rating for an elemental or compound CS never followed by the US and/or a negative US-expectancy rating for an elemental or compound CS always followed by the US on the very last trial of either elemental or compound training. These participants did not differ from the remaining sample on any of the demographic or personality variables. The conclusions of the experiment do not change when these participants are included in the analyses.

^2^Twelve percent of all trials across participants were not confirmed. The number of unconfirmed trials correlated negatively with the neuroticism scale of the EPQ, ρ(54) = −0.29, *p* = 0.04. Number of unconfirmed trials was not significantly related to any of the other questionnaire scores.
